# Adverse Pregnancy Outcomes in Sjogren's Disease Compared to Controls: An Interdisciplinary Approach with Maternal–Fetal Medicine

**DOI:** 10.1055/a-2562-1643

**Published:** 2025-04-08

**Authors:** Lauren Tesoriero, Jennifer Kidd, Julie Piccione, Peter Izmirly, Meredith Akerman, Steven Carsons, Patricia Rekawek, Julie Nusbaum

**Affiliations:** 1 Division of Rheumatology, Department of Internal Medicine, NYU Langone Hospital, Long Island, Mineola, New York; 2 NYU Long Island School of Medicine, Mineola, New York; 3 Northwell Health New Hyde Park, New York; 4 Donald and Barbara Zucker School of Medicine at Hofstra/Northwell Uniondale, Uniondale, New York; 5 Department of Obstetrics and Gynecology, Long Island Jewish Medical Center, New Hyde Park, New York; 6 Division of Rheumatology, NYU Langone Health, New York, New York; 7 NYU Grossman School of Medicine New York, New York; 8 Department of Maternal Fetal Medicine, NYU Langone Hospital, Long Island, Mineola, New York

**Keywords:** adverse pregnancy outcomes, Sjogren's disease, SS-A, SS-B antibodies, autoimmune disease

## Abstract

**Objectives:**

Outside of the association of SS-A antibody with congenital heart block, little is known about adverse maternal and neonatal outcomes, in patients with Sjogren's disease (SjD). Our study involved collaboration with maternal–fetal medicine (MFM).

**Methods:**

A retrospective cohort study of pregnant patients: SjD patients were matched 1:3 with non-SjD controls. SjD patients were included by meeting the 2016 ACR/EULAR Criteria or by a rheumatologist diagnosis. Exclusion criteria were concurrent autoimmune disease or related antibodies. A composite of grouped outcomes was utilized and verified by MFM specialists. The primary outcome was adverse pregnancy outcome (APO) between the two groups. Statistical analysis was performed using a two-sample *t* -test and Fisher's exact test.

**Results:**

48 patients were included: 12 SjD patients and 36 controls. APO was significantly increased in SjD with one preterm birth, one fetal growth restriction, and one limb anomaly; non-SjD had one cardiac anomaly. There were no cases of CHB. SjD patients were more likely to be delivered by cesarean delivery.

**Conclusion:**

There was an increased risk of APO in SjD patients compared with controls. No significant difference in neonatal outcomes was found. We speculate that placental pathology may play a role in pathophysiology and future studies should be performed.

**Key Points:**

- There was an increased risk of APO in SjD patients compared with controls.
- No significant difference in neonatal outcomes was found.
- We speculate that placental pathology may play a role in pathophysiology, prompting future studies.

## Introduction

Sjogren's disease (SjD) is an autoimmune disease primarily characterized by B cell infiltration of the lacrimal and salivary glands causing dry eye and dry mouth. The age-adjusted overall incidence and prevalence rates of SjD are 4.1 and 14.2 per 100,000 person-years with increased rates seen in females, which is six times higher than males. 1 Up to one-half of affected individuals also develop extra-glandular symptoms, including arthritis, myositis, Raynaud's syndrome, and cryoglobulinemia. 2 Pathogenesis of SjD manifestations remains incompletely understood. It is thought to be multifactorial, including both T cell (elevated levels of T Helper 1 cell cytokines and T Helper 17 cells reported in saliva of patients with SjD) and B cell (increased interferon type I activity) involvement. Major organ damage may occur in the setting of hyperglobulinemia leading to immune complex formation. 3

Outside of the known association of transplacental circulation of SS-A antibodies and congenital heart block (CHB) in 2% of exposed pregnancies, little is known about adverse maternal outcomes, and even less about neonatal outcomes. 4 Most of the current knowledge about neonatal and maternal risk in patients with SjD antibodies comes from research in patients who concomitantly have systemic lupus erythematosus (SLE), which can independently contribute to adverse maternal and neonatal outcomes. Few studies have evaluated pregnancy in patients exclusively with SjD, without concomitant autoimmune disease or related autoantibodies. A meta-analysis by Upala et al and a prospective study by Martin et al of pregnancy in this population have been conducted, but, to our knowledge, no studies have been performed in collaboration with maternal–fetal medicine (MFM) or have included significant information about perinatal outcomes. 5 6

Using a combined multidisciplinary approach between rheumatology and MFM, we aimed to characterize adverse maternal and neonatal outcomes in patients with SjD, without concomitant autoimmune diseases or related autoantibodies, and compare them with a matched control group. Our objective was to further our understanding of the maternal, obstetric, and neonatal risks associated with SjD in pregnancy.

## Methods

A retrospective cohort study was performed including pregnant patients with SjD, ages 18 to 60 years old, who had prenatal care and delivered at NYU Langone Hospital-Long Island (NYULH-LI) from January 2018 to December 2022. This timing corresponds to the initiation of Epic electronic health records at NYULH-LI and access to patient's complete medical history. Inclusion criteria for SjD patients were diagnosis by the 2016 ACR/EULAR Criteria or by rheumatologist evaluation (confirmed by comprehensive chart review by research investigators), with complete pregnancy data available. Exclusion criteria for SjD patients were concurrent autoimmune diseases such as SLE, antiphospholipid syndrome (APLS), rheumatoid arthritis, antibodies to dsDNA, Smith, or aPL; or unavailable pregnancy records. To select the control group, the AS OBGYN ultrasound program was utilized; this program is used at NYULH-LI by MFM physicians for fetal ultrasound imaging. The control group was reviewed by MFM research staff and matched to the SjD group based on the date of the anatomy ultrasound. The anatomy ultrasound date was utilized because it is routine for all pregnancies, including low-risk patients. Controls were also matched to maternal age (within ± 2 years of the SjD group) and gestational age (within ± 2 weeks of gestational age at the time of ultrasound). Of the limited population available for the control group, careful consideration was made in selecting patients without major maternal medical disease (such as pregestational diabetes and/or hypertension), surgical history, major fetal anomalies in pregnancy, prior adverse pregnancy outcome (APO), or delivery elsewhere. A total of three control patients were included for each patient with SjD so that statistical analysis could be completed in a matched comparison.

Many adverse maternal and neonatal outcomes of interest are rare, and so a composite of grouped outcomes was utilized. These composite outcomes were created in collaboration with the MFM department. The primary outcome was a composite of APO defined as miscarriage, intrauterine fetal death, fetal complication (intrauterine growth restriction and congenital anomalies [cardiac, lung, renal, neurologic, gastrointestinal, genitourinary, limb, hydrops, neural tube defect, thickened nuchal translucency]), fetal heart block (CHB), and preterm birth (gestational age <37 weeks). Secondary analysis included the following composites: maternal hypertensive outcomes, maternal infectious outcomes, and other adverse outcomes (antepartum, during delivery, and in the neonate).

Statistical analysis of maternal demographics, maternal outcomes, and neonatal outcomes was performed using a two-sample *t* -test for continuous variables and Fisher's exact test for categorical variables, with a significance *p* -value of < 0.05. Descriptive statistics (mean ± standard deviation for continuous variables; frequencies and percentages for categorical variables) were calculated separately by group (Sjogren's cases vs. non-Sjogren's controls). All analyses were performed using SAS version 9.4 (SAS Institute Inc., Cary, NC).

## Results

Twelve patients with SjD and 36 non-SjD controls were included in our analysis. SjD patients were more likely to be prescribed Aspirin (50% vs. 5.6%, *p* = 0.002) and Hydroxychloroquine (33.33% vs. 0%, *p* = 0.003), while other demographics were comparable between the groups. There were no significant differences among the number of patients with advanced maternal age at birth (defined as greater than or equal to 35 years old, *p* = 0.87), and prepregnancy medical conditions were rare with only 1 SjD patient and 1 control with hyperlipidemia, *p* = 0.44. Of those with SjD disease, SS-A/SS-B antibody positivity status was analyzed and 4/12 (33.33%) were SS-A only, 2/12 (16.67%) were SS-B only, 4/12 (33.33%) were SS-A and SS-B, and 2/12 (16.67%) were neither SS-A nor SS-B positive. Of the 2 patients who were neither SS-A nor SS-B positive, both received diagnoses based on the presence of sicca symptoms. One patient had antibodies to salivary gland protein 1 and 1 patient had a history of previously testing positive for SS-A and SS-B antibodies while they were under the care of a different rheumatologist. In total, 7/12 (58.3%) were ANA positive and 2/12 (16.7%) were RF positive ( Table 1 ). None of the patients received a biopsy.

**Table 1:** Demographics

Variable	Patients with SjD ( *n* = 12)	Non-SjD controls ( *n* = 36)	*p* -Value
Age (y) a	35.58 ± 4.44	34.19 ± 4.20	0.33
Advanced maternal age at birth			0.87
≥35 y	6 (50.00%)	19 (52.80%)	
Body mass index (kg/m ^2^ ) a	27.56 ± 4.28	27.07 ± 6.07	0.79
Race			0.67
Asian	1 (8.33%)	1 (2.78%)	
Black/African American	1 (8.33%)	7 (19.44%)	
White	6 (50.00%)	18 (50.00%)	
Unknown/other	4 (33.33%)	10 (27.78%)	
Ethnicity				0.54
Non-Hispanic	6 (50.00%)	24 (66.67%)		
Hispanic/Latino	4 (33.33%)	8 (22.22%)		
Unknown	2 (16.67%)	4 (11.11%)		
Prepregnancy condition			
Hypertension	0 (0.00%)	0 (0.00%)	NA
Diabetes	0 (0.00%)	0 (0.00%)	NA
Hyperlipidemia	1 (8.33%)	1 (2.78%)	0.44
Coronary artery disease	0 (0.00%)	0 (0.00%)	NA
Chronic kidney disease	0 (0.00%)	0 (0.00%)	NA
In vitro fertilization	1 (8.33%)	4 (11.11%)	1
Multiparous	9 (75.00%)	26 (72.22%)	1
Medications in pregnancy			
Aspirin	6 (50.00%)	2 (5.56%)	0.002
Hydroxychloroquine	4 (33.33%)	0 (0.00%)	0.003
Oral corticosteroids	0 (0.00%)	0 (0.00%)	NA
SjD antibody positivity			
SS-A only	4 (33.33%)		
SS-B only	2 (16.67%)		
SS-A and SS-B	4 (33.33%)		
Neither	2 (16.67%)		
Other antibodies			
ANA	7 (58.33%)		
RF	2 (16.67%)		

Abbreviation: SjD, Sjogren's disease.

a Reported as mean ± standard deviation.

Note: All outcomes are reported at *n* (percent) unless otherwise noted below.

All patients in both the SjD and control group had live births with no miscarriages or occurrence of intrauterine fetal demise. SjD patients were significantly more likely to deliver by cesarean section (75%) as compared with controls (25%), *p* < 0.01. The indications for cesarean section among SjD patients were: five repeat Caesarean sections, two elective Caesarean sections, one arrest of dilation, and one arrest of descent and fetal intolerance. The average birth weight was similar between groups 3,308 and 3,404 g ( *p* = 0.6) with no neonates born weighing less than 2,500 g ( Table 2 ).

**Table 2:** Delivery outcomes

Variable	Patients with SjD ( *n* = 12)	Non-SjD controls ( *n* = 36)	*p* -Value
Live birth rate	12 (100.00%)	36 (100.00%)	NA
Miscarriage	0 (0.00%)	0 (0.00%)	
Intrauterine fetal demise	0 (0.00%)	0 (0.00%)	
Mode of delivery			<0.01
Vaginal	2 (16.67%)	25 (69.44%)	
Operative	1 (8.33%)	2 (5.56%)	
Cesarean	9 (75.00%)	9 (25.00%)	
Induction of labor	4 (33.33%)	20 (55.56%)	0.18
Gestational age at birth (wks) a	38.63 ± 1.48	39.53 ± 0.96	0.13
Preterm birth (prior to 37 wks)	1 (8.33%)	0 (0.00%)	0.25
Birth weight (g) a	3,308 ± 455	3,404 ± 332	0.60
Intrauterine growth restriction	1 (8.33%)	0 (0.00%)	0.25
Birthweight less than 2,500 g	0 (0.00%)	0 (0.00%)	NA
Umbilical cord pH (arterial blood gas) a	7.25 ± 0.05	7.24 ± 0.09	0.97

Abbreviation: SjD, Sjogren's disease.

a Reported as mean ± standard deviation.

Note: All outcomes are reported at *n* (percent) unless otherwise noted below.

SjD patients were significantly more likely to have an increased APO as compared with non-SJD controls (25% vs. 2.8% *p* = 0.04; Table 3 ). The APO outcomes for SjD patients included 1 preterm birth, 1 case of fetal growth restriction, and 1 fetal limb anomaly ( Table 4 ). There was 1 patient in the control group with an associated atrial septal defect ( Table 4 ). There were no cases of CHB in either group. Regarding secondary outcomes, there was a trend toward increased maternal hypertensive outcomes among patients with SjD compared with controls (25% vs. 8.33%), however this with not statistically significant ( *p* = 0.156). There were no other significant differences in adverse maternal or neonatal outcomes ( Table 3 ).

**Table 3:** Composite outcomes

Composite outcomes	Patients with SjD ( *n* = 12)	Non-SjD Controls ( *n* = 36)	*p* -Value
Adverse pregnancy a	3 (25.00%)	1 (2.78%)	0.040
Maternal infectious b	0 (0.00%)	0 (0.00%)	NA
Maternal hypertensive c	3 (25.00%)	3 (8.33%)	0.156
Adverse delivery d	2 (16.67%)	7 (19.44%)	1.000
Adverse maternal antepartum e	2 (16.67%)	3 (8.33%)	0.587
Adverse neonatal f	0 (0.00%)	4 (11.11%)	0.560

Abbreviation: SjD, Sjogren's disease.

a Adverse pregnancy composite: miscarriage, intrauterine fetal death, fetal complication (intrauterine growth restriction and congenital anomalies [cardiac, lung, renal, neurologic, gastrointestinal, genitourinary, limb, hydrops, neural tube defect, thickened nuchal translucency]), fetal heart block, and preterm birth (gestational age <37 weeks).

b Adverse infectious composite: chorioamnionitis, endometritis, mastitis, and wound infection.

c Maternal hypertensive composite: gestational hypertension, preeclampsia, eclampsia, HELLP (hemolysis, elevated liver enzyme, and low platelet) syndrome, and postpartum preeclampsia.

d Adverse delivery composite: third/fourth-degree laceration, episiotomy, cesarean delivery for fetal intolerance of labor, postpartum hemorrhage, shoulder dystocia, placenta accreta spectrum, retained placenta, blood transfusion, hysterectomy, intensive care unit admission, and maternal death.

e Adverse maternal antepartum composite: venous thromboembolism, gestational diabetes, vasa previa, placenta previa, and placental abruption.

f Adverse neonatal composite: neonatal intensive care unit, neonatal complications (infection [sepsis, pneumonia, neonatal necrotizing enterocolitis], respiratory problem [mechanical ventilation, RDS], neurologic [seizures, hypoxic-ischemic encephalopathy, intraventricular hemorrhage]), neonatal demise, birthweight less than 2,500 g, and umbilical cord arterial blood gas pH <7.0, APGAR (appearance, pulse, grimace, activity, and respiration) 5-minute score <7.

Note: All outcomes are reported as *n* (percent).

**Table 4:** Patients with adverse pregnancy outcomes

	Patient A	Patient B	Patient C	Patient D
Diagnosis of SjD?	Yes	Yes	Yes	No
SS-A positivity (titer)	Yes	No	No	NA
SS-B positivity (titer)	Yes	No	Yes	NA
Worsening symptoms?	Unknown	Yes—dry eyes, dry mouth, joint pain	Unknown	NA
Medications	Prenatal vitamin	Prenatal vitamin	Prenatal vitamin	Prenatal vitamin
Comorbidities	Anemia	Anxiety, depression, bipolar	Anxiety	Anemia
Ethnicity	Non-Hispanic	Non-Hispanic	Non-Hispanic	Hispanic/Latinx
Race	Black	White	White	Other
Alcohol/tobacco/drugs	None	None	None	None
Maternal complication(s)	Preeclampsia, endometritis	None	None	None
Fetal complication(s)	Preterm	IUGR	Fetal limb anomaly, genetic aneuploidy	ASD

Abbreviation: SjD, Sjogren's disease.

## Discussion

Patients with SJD were at increased risk of APO in our study as compared with those without SjD. Although there was an increased risk of APO in SjD patients, there were no significant differences in adverse neonatal outcomes, which is reassuring. As prior research has mainly focused on CHB associated with SS-A antibodies in SjD, the diverse outcomes analyzed in our study contribute substantially to the body of research exploring pregnancies with autoimmunity. 4

From our understanding, few studies have been performed to date to evaluate pregnancy outcomes in patients with SjD, in the absence of other autoimmune diseases. Our study excluded patients diagnosed with an additional autoimmune disease and any patient with a related positive autoantibody. This rigorous study criterion allowed for the analysis of patients with “pure SjD” (formerly known as primary SjD). Much of what we know about adverse maternal and neonatal outcomes in patients with autoimmune disease comes from studies conducted in SLE and APLS. Maternal mortality is 20-fold higher among women with SLE with three- to sevenfold higher rates in thrombosis, infection, thrombocytopenia, and transfusion. 7 SLE patients also have a higher risk of cesarean section, preterm delivery, and preeclampsia. 7 In a retrospective study from California Health Information, Yasmeen et al found increased rates of hypertensive complications, renal disease, preterm delivery, unplanned cesarean delivery, postpartum hemorrhage, and maternal venous thromboembolism in the SLE population. 8 In addition, they found neonatal and fetal outcomes were significantly worse in the SLE group, with higher rates of fetal growth restriction and neonatal death. 8 Among patients with APLS, the most common adverse maternal outcomes are recurrent pregnancy loss, preeclampsia, placental insufficiency, maternal thrombosis (including stroke), and complications due to treatment. 9 Common neonatal outcomes in women with APLS include preterm birth, IUGR, and intrauterine fetal demise. 10 The PROMISSE study, which enrolled pregnant women with SLE and/or aPL antibodies and pregnant health controls, found that increased levels of Bb and sC5b-9 in early pregnancy were strongly predictive of APO and suggested a pathogenic role of the alternative pathway. 11

The strengths of our study include the interdisciplinary approach with MFM and the use of rigorous definitions of maternal, obstetric, and neonatal adverse outcomes. In addition, we analyzed patients with only SjD and excluded those with co-existing autoimmune conditions or related autoantibodies to limit the potential for confounding.

Limitations are those inherent to any retrospective study. Our study was limited by a small sample size, and our maternal demographics may not be generalizable to other institutions. In addition, there was the potential for confounding as multivariable logistic regression analysis was not performed due to the small sample size. In particular, as SjD was significantly associated with an increased rate of maternal Aspirin and Hydroxychloroquine use, future prospective studies with larger numbers are needed to control for this potential confounder. Information regarding the patient's symptoms was not complete enough to evaluate disease activity during pregnancies. This would be a consideration for evaluation in further studies.

Defining SjD in research is challenging. In this study, we opted to include patients who met ACR-EULAR criteria or who were given the diagnosis of SjD by a rheumatologist evaluation. This allowed us to capture the most accurate patient population based on the data available in the electronic health record. The authors were consistent with the study criteria; however, the original electronic health record could not be fully reflective of the patient's history, management, and outcome.

It is unclear why there are more APOs among patients with SjD, but there are several biological possibilities. Autoimmunity in general, the presence of pro-inflammatory cytokines and complement activation may have a role in embryo implantation and placental development. Complement activation can cause damage in fetal tissues, as this occurs in some known APOs such as recurrent spontaneous abortions and preeclampsia. 12 Based on our immunological understanding of pregnancy in other diseases like SLE and RA, we can speculate that placental pathology (decidual vasculopathy, chronic inflammation, inflammatory lesions) 13 may play a role in APO in the SjD population. However, our study does not explain this causality and calls for further exploration. Future studies should be performed analyzing placental histopathology in those with SjD as compared with those without.

Our study adds to the limited knowledge of maternal, obstetric, and neonatal outcomes associated with SjD. As SjD is associated with a higher risk of APO, clinicians should consider closer monitoring and early collaboration with MFM specialists in the management of these pregnancies.
